# A computational model for predicting the transport of compounds by ABCC2

**DOI:** 10.1186/1758-2946-4-S1-P53

**Published:** 2012-05-01

**Authors:** Marta Pinto, Gerhard F Ecker

**Affiliations:** 1 Department of Medicinal Chemistry, University of Vienna, Vienna, 1090, Austria

## 

The ATP-binding cassette (ABC) proteins represent a large family of transmembrane proteins that use the energy of ATP hydrolysis to transport a wide variety of physiological substrates across biological membranes [1]. Of them, particular attention has been focused in the last years on the role of the ABCC2 transporter in drug clearance and disposition.

The ABCC2 transporter is a transmembrane protein expressed in the apical cell membrane of hepatocytes and epithelial cells of small intestine and kidney, where it is involved in the elimination of many endogenous and exogenous substrates from the cell, including compounds clinically relevant [2]. Alteration in the disposition and elimination of these compounds can modify their pharmacokinetic and pharmacological profiles, leading to reduced efficacy or increased toxicity.

In this scenario, the aim of the present work was the development of an *in silico* model based on the Gottesman database [3] able to predict if certain compounds of interest are ABCC2 substrates or not. To this end, several machine learning methods were explored using the WEKA data mining software [4]. Molecules were represented by 2D and 3D descriptors calculated with the MOE software [5]. Feature selection was used to improve the efficiency of the data mining algorithms and identify the contribution of different features. Misclassification cost was used in order to deal with data set imbalance. According to our results, naive Bayesian updatable (NBU) had the highest performance, with an overall prediction accuracy of 72.1% and a Matthew’s correlation coefficient of 0.44. Furthermore, sensitivity and specificity values were significantly improved with values of 72.7% and 71.6%, respectively.
